# *FGF-2* Gene Polymorphism in Osteoporosis among Guangxi’s Zhuang Chinese

**DOI:** 10.3390/ijms18071358

**Published:** 2017-06-27

**Authors:** Xiaoyun Bin, Chaowen Lin, Xiufeng Huang, Qinghui Zhou, Liping Wang, Cory J. Xian

**Affiliations:** 1 College of Basic Medical Sciences, Youjiang Medical University for Nationalities, Baise 533000, China; bxy889@163.com (X.B.); lcwhxf@163.com (C.L.); zqh2853495@163.com (Q.Z.); 2 Sansom Institute for Health Research and School of Pharmacy and Medical Sciences, University of South Australia, Adelaide, SA 5001, Australia; liping.wang@mymail.unisa.edu.au

**Keywords:** bone ultrasound, osteoporosis, human association studies, fibroblast growth factor-2, aging

## Abstract

Osteoporosis is a complex multifactorial disorder of gradual bone loss and increased fracture risk. While previous studies have shown the importance of many genetic factors in determining peak bone mass and fragility fractures and in suggesting involvement of fibroblast growth factor-2 (*FGF-2*) in bone metabolism and bone mass, the relationship of *FGF-2* genetic diversity with bone mass/osteoporosis has not yet been revealed. The current study investigated the potential relevance of *FGF-2* gene polymorphism in osteoporosis among a Zhuang ethnic Chinese cohort of 623, including 237 normal bone mass controls, 227 osteopenia, and 159 osteoporosis of different ages. Bone density was examined by calcaneus ultrasound attenuation measurement, and single nucleotide polymorphisms (SNPs) and linkage disequilibrium analyses were performed on five SNP loci of *FGF-2* gene. Significant differences were found in bone mass in males between the 45-year-old and ≥70-year-old groups (*p* < 0.01), and in females among 55, 60, 65 and 70-year-old groups (*p* < 0.05). Males had higher bone mass values than females in the same age (over 55-year-old) (*p* < 0.05). The proportions of individuals with normal bone mass decreased with age (65.2% to 40% in males, and 50% to 0% in females), whereas prevalence of osteoporosis increased with age (15.4% to 30% in men, and 7.7% to 82% in women). Out of five *FGF-2* SNP loci, the TA genotype of rs308442 in the osteoporosis group (40.2%) was higher than in the control group (29.5%) (*p* < 0.05). The TA genotype was significantly correlated with the risk of osteoporosis (odds ratio OR = 1.653), 95% confidence interval (CI): 1.968–1.441). Strong linkage disequilibrium in *FGF-2* gene was also detected between rs12644427 and rs3747676, between rs12644427 and rs3789138, and between rs3747676 and rs3789138 (D’ > 0.8, and *r*^2^ > 0.33). Thus, the rs308442 locus of *FGF-2* gene is closely correlated to osteoporosis in this Zhuang ethnic Chinese cohort, and the TA may be the risk genotype of osteoporosis.

## 1. Introduction

Osteoporosis is a systemic skeletal disease characterized by a progressive reduction in bone mass and deterioration of the bone architecture and strength, resulting in an elevated risk of fracture [1]. The incidence of osteoporosis is influenced by heredity, environment, gender, age, nutrition, life style, physical exercise, drug use, disease, and various other factors. Among these factors, genetics accounts for 60–85% of the influence [2]. Moreover, the occurrence of osteoporosis and osteoporotic fracture depends on peak bone mass obtained and bone loss rate, both of which are also influenced by the aforementioned factors [3].

Previously, many genetic studies used the candidate gene approach to explore the genes associated with osteoporosis [4,5,6]. For example, macrophage colony-stimulating factor, receptor activator of nuclear factor-κB ligand, osteoprotegerin, interleukin-34, and other factors have been proven to participate in the osteoclast differentiation through activation of the non-canonical Wnt pathway [4,5,6]. Also demonstrating a key role of the canonical Wnt pathway in determining bone mass, it was shown that β-catenin deletion led to a decreased number of osteoblasts, an increased number of osteoclasts, and decreased bone mass [7,8]. Furthermore, bone morphogenetic protein (BMP) signaling pathway is also critical for cartilage and bone formation and postnatal bone development and regulation of bone metabolism [9]. Due to their critical roles in regulating bone mass, these factors are either currently and/or likely to be used as drug targets to intervene with the occurrence and development of osteoporosis. However, further studies are required to identify other likely genetic factors for osteoporosis.

Fibroblast growth factor (FGF-2) is a growth factor produced by and released from epithelial and mesenchymal cells. *FGF-2* can regulate normal cell division, proliferation, migration, and differentiation [10]. Moreover, FGF/FGF receptor (FGFR) signaling has been found to be an important pathway in skeletal development [11]. Lei et al. [12] showed that *FGF-2* can promote osteogenic differentiation of cultured human bone marrow mesenchymal stem cells. Naganawa et al. [13] exhibited that bone formation in *FGF-2* gene knockout mouse was obviously decreased. Nagayasu-Tanaka et al. [14] proved the role of *FGF-2* in bone formation and osseointegration. While evidence above confirmed that *FGF-2* plays an important role in bone formation and bone mass determination, the relationships between *FGF-2* genetic diversity with bone mineral density (BMD) and with risk of osteoporosis have not yet been revealed.

Since FGF-2 is known to be important in regulating skeletal development and formation of osteoblasts [15], and previously, Hao et al. [16] observed relationships of plasma FGF-2 levels and polymorphism of *FGF-2* gene with the obese phenotype of the Chinese Han population, in the current study, we speculated that *FGF-2* gene polymorphism and its haplotypes may be related to the occurrence and development of osteoporosis. To determine whether *FGF-2* can be used as a genetic marker to predict the risk of osteoporosis in middle-aged and elderly people, the relationship between *FGF-2* haplotypes and bone mass was explored, and the distribution of *FGF-2* gene polymorphism and its haplotypes in osteoporosis patients and normal bone mass people were analyzed in middle-aged and senior Zhuang ethnic people in Guangxi, a multi-ethnic region of China.

## 2. Results

### 2.1. The Bone Mass Decreasing Trends of the Male and Female Groups with Age

**Figure 1:**
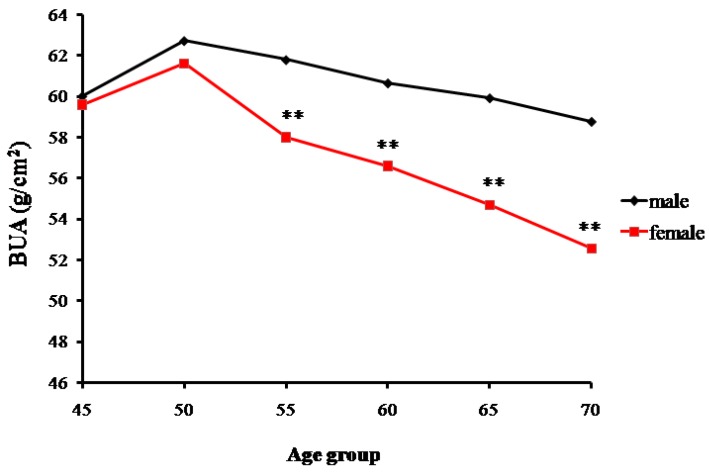
Changes in the bone mass density (assessed as broadband ultrasound attenuation or BUA) with age of senior and middle-aged Zhuang people in Guangxi (** *p* < 0.01 versus males).

Data for the measured broadband ultrasound attenuation (BUA) showed a gradual declining trend of bone mass with increasing age. The trends of changes in bone mass in senior and middle-aged Zhuang men and women in Guangxi are shown in Table 1 and Figure 1. A significant difference was found in the male BUA between the 45-year-old and ≥70-year-old groups (*p* < 0.01), but no statistical differences were found among the 50, 55, 60, and 65-year-old groups (*p* > 0.05). Similarly, BUA values of women gradually decreased with age. Significant differences were found in the BUA values between the ≥70-year-old group and the other age groups (*p* < 0.01). Additionally, significant differences were found between the male and female groups, respectively, for the 55, 60, 65, and 70-year-old groups (*p* < 0.01). However, for the 45 and 50-year-old groups, males and females had the similar BUA values.

**Figure 2:**
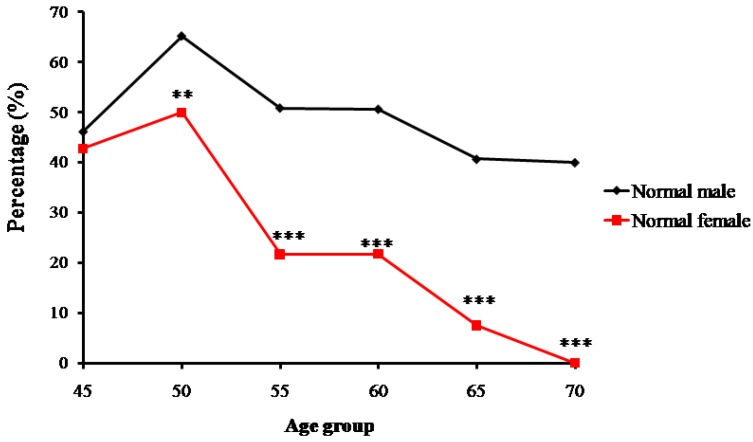
Trends of percentage changes with age in individuals with normal bone mass among senior and middle-aged Zhuang people (** *p* < 0.01 versus males, *** *p* < 0.001 versus males).

**Figure 3:**
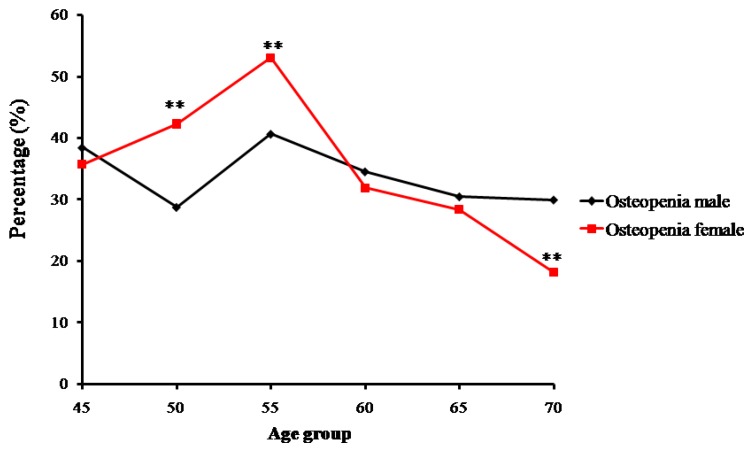
Trends of percentage changes with age in individuals with osteopenia among senior and middle-aged Zhuang people (** *p* <0.01 versus males).

**Figure 4:**
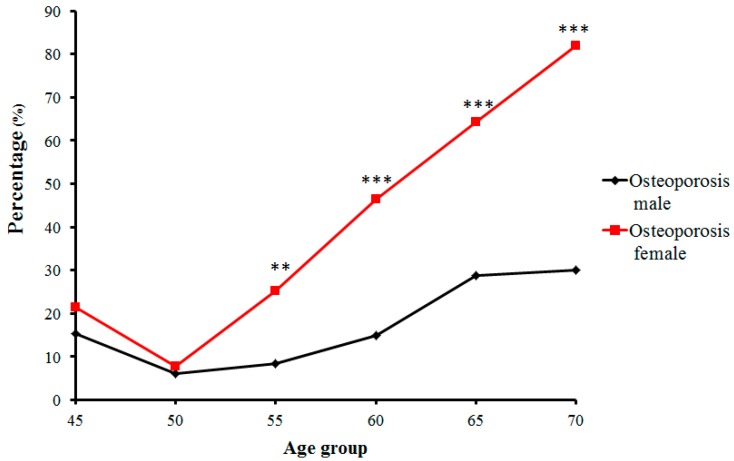
Trends of percentage changes with age in individuals with osteoporosis among senior and middle-aged Zhuang people (** *p* < 0.01 versus males, *** *p* < 0.001 versus males).

Trends of percentage changes in individuals with normal bone mass, osteopenia, and osteoporosis among the senior and middle-aged Zhuang people in Guangxi are shown respectively in Figure 2, Figure 3 and Figure 4. The age-related changes in numbers and prevalence of individuals with normal bone mass, osteopenia, and osteoporosis are shown in Table 2. As shown in Figure 2, the number and proportion of Zhuang people with normal bone mass gradually decreased as age increased for both men and women. The proportion of males with normal bone mass in the 45-year-old group was 46.2%, which then increased to 65.2% in the 50-year-old group. From the 55-year-old group to the 70-year-old group, the proportion gradually decreased from 50.9% to 40%. The relatively small sample size of the 45-year-old group may have led to the higher percentage of individuals with normal bone mass in the 50-year-old group than the 45-year-old group.

The proportion for females with normal bone mass in the 45-year-old group was 42.7%, which increased to 50% in the 50-year-old group. It then decreased from 21.7% in the 55-year-old group to 0% in the 70-year-old group. Again, the relatively small sample size of the 45-year-old group may have led to the lower percentage of individuals with normal bone mass than the 50-year-old group (Figure 2).

In contrast, the number and proportion of Zhuang people with osteopenia and osteoporosis gradually increased. Proportions of osteopenia fluctuated from 28.8% (in the 50-year-old group) to 40.7% (in the 55-year-old group) in males, and from 53.0% (in the 55-year-old group) to 18.2% (in the 70-year-old group) in females (Figure 3). Prevalence of osteoporosis in Zhuang men increased with age from 15.38% (in the 45-year-old group) to 30% (in the 70-year-old group) (*p* < 0.01), whereas that in Zhuang women increased from 7.69% (in the 50-year-old group) to 81.81% (in the 70-year-old group). Furthermore, while no strict changes in proportions of normal bone mass, osteopenia, or osteoporosis were found between adjacent age groups, the tendency was roughly consistent with the change of age for all groups (Figure 4) (*p* < 0.01) (Table 2).

### 2.2. FGF-2 Genotype and Allelic Gene Frequency Distribution

The detection of five SNP loci of *FGF-2* gene shows the polymorphism of five polymorphic loci of *FGF-2* gene existing in the Zhuang people of Guangxi, China. The distributions of genotypes and allelic gene for all polymorphic loci of the *FGF-2* gene are shown in Table 3. The five polymorphic loci (rs308379, rsl2644427, rs3789138, rs308442, and rs3747676) of the *FGF-2* gene reached the Hardy-Weinberg equilibrium through χ^2^-test (*p* > 0.05). The distribution frequency of the TA genotype in the rs308442 of the *FGF-2* gene in the normal group (50 cases) was significantly lower than that in the Osteopenia group (84 cases) or that in Osteoporosis group (91 cases) (*p* < 0.05). Furthermore, the TA genotype of rs308442 was significantly correlated with the risk of osteoporosis (Normal vs. Osteoporosis: odds ratio, OR = 1.652, 95% confidence interval, CI: 1.960–1.424, *p* = 0.031; Normal vs. Osteopenia: OR = 1.653, 95% CI: 1.968–1.441, *p* = 0.033). No statistically significant differences were found in the distributions of genotypes and allelic gene of the remaining four polymorphic loci of *FGF2* gene (rs308379, rsl2644427, rs3789138, and rs3747676) (*p* > 0.05) (Table 3).

The bone mass density of TA genotype (58.75 ± 5.45) in the rs308442 of the *FGF-2* gene was lower than that of TT (61.04 ± 5.81) or AA (60.59 ± 5.55) genotype (*p* < 0.05). However, no statistically significant differences were found within genotypes in the other four *FGF-2* SNP loci (rs308379, rsl2644427, rs3789138, and rs3747676) (*p* > 0.05) (Table 4).

### 2.3. Linkage Disequilibrium (LD) Analyses

Haploview 4.2 free software (from http://www.broadinsti tute.org/haploview/haploview-downloads) was used to simulate LD for the five SNP loci of the detected *FGF-2* gene. LD coefficients D’ and *r*^2^ between *FGF-2* mutant loci are shown in Table 5 and Table 6, respectively. The results showed the LD coefficients of D’ > 0.8 and *r*^2^ > 0.33 between rs12644427 and rs3747676, between rs12644427 and rs3789138, and between rs3747676 and rs3789138 of *FGF-2* gene. These results indicate a strong LD. The LD coefficient between the remaining two loci did not meet the Hardy-Weinberg equilibrium, which indicates a lack of strong LD (D’ < 0.8 or *r*^2^ < 0.33). The LD types between the case and control groups were almost the same.

**Figure 5:**
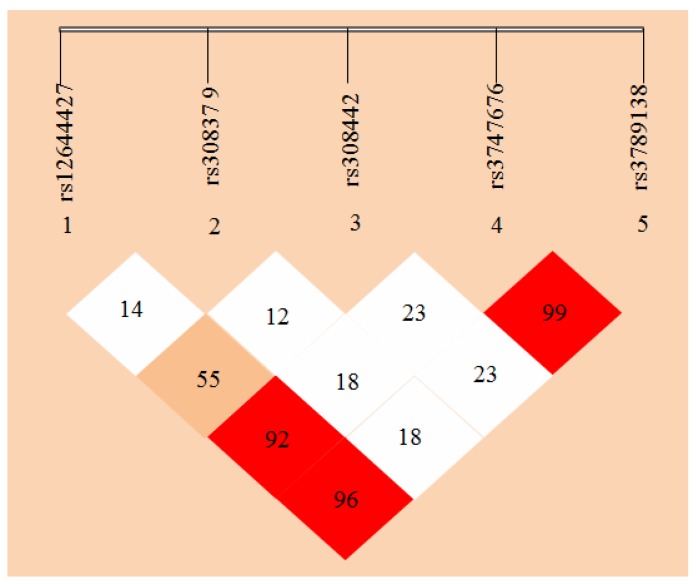
*FGF-2* gene linkage disequilibrium and haplotype block structure. Values in cells indicate logarithm of odds (LOD) score of D’.

*FGF-2* gene LD and haplotype block structure are shown in Figure 5 with values in the cells indicating the genetic linkage logarithm of odds (LOD) score of D’. These results show that five SNPs of *FGF-2* gene are in incomplete linkage disequilibrium, and there is no haplotype is constituted (Table 5 and Table 6).

## 3. Discussion

It is now believed that 50–85% of the variance in BMD and risks of osteoporosis is controlled by genetic factors which are mostly polygenic [2]. Guangxi of China is a multi-ethnic region, and the study on gene polymorphism of its population is very important to explore the distribution characteristics of pathogenic and susceptibility genes of osteoporosis and to study osteoporosis on the pathogenesis and gene levels. In this study, the trends of age-related changes of bone mass (as estimated by calcaneus ultrasound attenuation) were analyzed among senior and middle-aged Zhuang ethnic people in Guangxi. In addition, differences in the *FGF-2* gene polymorphism in five SNP loci between osteoporosis patients and individuals with normal bone mass were assessed, and the linkage disequilibrium (LD) of *FGF-2* in the five SNP loci was evaluated. The current study has shown a clear relationship between *FGF-2* gene polymorphism and bone mass, and by investigating SNPs and haplotype of *FGF-2* gene and the risk of osteoporosis, *FGF-2* gene was shown to be potentially useful genetic marker for predicting osteoporosis risk in senior and middle-aged Zhuang people.

### 3.1. Age-Related Changing Trends of Bone Mass

Calcaneus quantitative ultrasound measurement has become an internationally recognized method for bone health examination and osteoporosis/fracture risk prediction. This process is noninvasive, nonradioactive, easy to operate, low cost, and precise. Calcaneus quantitative ultrasound measurement can be used to fully reflect bone mass, bone structure, and bone properties, because almost all cancellous substances are sensitive to changes in bone mass. Moreover, several studies have proved that calcaneus ultrasound parameters can sensitively reflect the changes in bone mineral and collagen contents [17]. Calcaneus ultrasound can be used as an effective tool for bone health survey [18] and prediction of fracture risk of senior and middle-aged people [19]. In this study, bone mass was measured using calcaneus quantitative ultrasound, which can objectively reflect the trends of changes in population bone density with age.

The effect of aging on BMD is irreversible [20]. Adammi et al. studied the relationship between bone density with age, body weight, and life style and found a significant negative correlation between bone and age [21]. Evans also verified that the bone density in normal weight and obese population decreased with age [22]. In the current study, bone density in the senior and middle-aged Zhuang people in Guangxi showed a gradual decline with age. While the decline in males was found to be smooth and gradual, significant bone mass differences were found only between the ≥70-year-old group and 45-year-old group, although the differences among the other age groups of males were not significant. Thus, the bone mass of this male population started to significantly decrease from 70 years old, and this age group would later have a low bone density. This phenomenon is believed to be due to the fact that local males maintain long outdoor labor time until the senior age, as they are the major labor force of the family.

The decreases in BMD in the senior and middle-aged Zhuang females were found to be larger and to occur faster. In particular, bone density of the ≥50-year-old females decreased sharply. The proportions of individuals with normal bone mass decreased from 50% for the 50-year-old group to 0% for the 70-year-old group, while proportions of osteoporosis increased correspondingly from 7.69% to 81.82%. This sharp decrease in bone mass is believed to be due to the fact that most females aged 55 have been in menopause, which is characterized with a sudden decrease in estrogen secretion. Following menopause, bone resorption rate is known to be greater than bone formation rate, and bone loss becomes considerable, reaching 2.0–3.0% annually, compared to the annual bone loss caused by aging being approximately 1.0% (present in both males and females) [23]. After 70 years of age, the decrease in bone density was more evidently affected by the estrogen deficiency and aging. Male bone density, on the other hand, gradually decreased with age, and its declining trend was smoother and slower than that of females. Donner et al. [24] investigated the impact of androgenic hormone on bone density using rats and showed that age-related testosterone deficiency can cause femoral osteoporosis of experimental animals, as well as adverse bone geometry and impaired bone strength in male rats. These conclusions are consistent with the results obtained in the present study.

### 3.2. Distribution of FGF-2 Gene SNPs in Zhuang Osteoporosis Patients and Normal Bone Mass Individuals

Since the cellular growth factor *FGF-2* is known to be important in skeletal development and metabolism due to its effects in chemotaxis, mitosis, in the formation of chondrocytes and osteoblasts, and in angiogenesis [16,25,26,27,28], in the current study, we speculated that *FGF-2* gene polymorphism and its haplotypes may be related to the occurrence of osteoporosis. The *FGF* gene is located at the 4q26-4q27 chromosome, which is composed of 71.529 kb base, including three exons and two introns. At present, 8253 SNPs of *FGF-2* have been verified according to the NCBI database (https://www.ncbi.nlm.nih.gov/). *FGF-2* gene polymorphism has been proved to be significantly associated with the occurrence of various diseases or cancers. Hao et al. [16] observed relationships of plasma *FGF-2* levels and polymorphism of *FGF-2* gene with the obese phenotype of Chinese Han population. Wang et al. [29] found that one SNP locus rs11737764 of the *FGF-2* gene is significantly associated with the increase of osteosarcoma susceptibility. Kang et al. found that *FGF-2* 754C/G polymorphic loci are significantly associated with the occurrence of ectopic endometrium, and people with G allelic gene have a low risk of the disease [30]. Slattery et al. demonstrated that the combined action of *FGF-2* gene polymorphic loci and growth factor pathway-related genes can affect the risk of breast cancer [31]. Some studies have reported that *FGF-2* gene SNPs, namely, rs6854081, rs1048201, and rs7683093, are significantly associated with bone density of collumfemoris. Moreover, three SNP loci (rs6854081, rs1048201, and rs7683093) of *FGF-2* gene have been proved to be closely related to the bone mass density of the femur neck [32]. The increase of expression in *FGF-2* in the bone has been suggested to promote the occurrence and development of osteoporosis and is associated with pathogenesis of osteoporosis [11].

Analyses of the distributions of five SNPs of the *FGF-2* gene and its genotype in Zhuang osteoporosis patients and normal bone mass individuals showed that five SNPs of the *FGF-2* gene, namely, rs308379, rsl2644427, rs3789138, rs308442, and rs3747676, reached Hardy-Weinberg equilibrium by χ^2^-test (*p* > 0.05). This result indicates that the distributions of various groups of genotypes of many loci of *FGF-2* gene in the studied populations have reached genetic equilibrium. Among the five SNPs of the *FGF-2* gene, the distribution frequency of the only mutant genotype TA in rs308442 locus in the case group (40.2%) was higher than that in the control group (29.5%). Our data suggests that TA is a risk factor of osteoporosis (OR = 1.531, 95% CI: 2.179–1.078) (Table 3). The differences in genotype frequency and allelic gene frequency of the remaining four loci of *FGF-2* gene, namely, rs308379, rsl2644427, rs3789138, and rs3747676, between the case and control groups showed no statistical significances (*p* > 0.05).

The results are consistent with the study of Dong et al. on the correlation between the bone density of collumfemoris and *FGFR-2* gene polymorphism in the Han population [33]. They showed that two of the 28 SNPs of the *FGFR-2* gene (i.e., rs11200014 and rs1078806) are significantly associated with bone density of collumfemoris. Furthermore, analyses showed that *FGF-2* and its receptor *FGFR-2* are similarly associated with bone density. Thus, based on these previous studies and our current data, *FGF-2* signal pathway participates in the occurrence and development of osteoporosis.

### 3.3. Linkage Disequilibrium (LD) Analyses of the SNPs of FGF-2 Gene

D’ and *r*^2^ are two common parameters for assessing LD. The probability of the occurrence of recombination events in the LD area can be directly reflected with the value of D’, and the validity of association analysis is directly related to *r*^2^. Several studies have suggested that if |D’| > 0.8, then two loci are in strong LD. If *r*^2^ > 0.33, two SNPs are closely linked to be inherited as a whole [34,35]. However, D’ is relatively insensitive to the changes in gene frequency, and when a gene frequency in a locus is lower, *r*^2^ is more reliable than D’ [36].

In this study, to determine the association of *FGF-2* SNPs with osteoporosis, LD analyses were performed by pairs of the five polymorphic loci of *FGF-2*, and D’ and *r*^2^ were comprehensively analyzed. Strong LDs were shown between rs12644427 and loci rs3747676 and rs3789138, and between rs3747676 and rs3789138 (D’ > 0.8 and *r*^2^ > 0.33). These results indicate the likely linkage units among three SNP loci, namely, rs12644427, rs3747676, and rs3789138. However, no correlation was found between the polymorphism of rs12644427, rs3747676, rs3789138, and osteoporosis, and locus rs308442 (which is associated with osteoporosis) has no linkage relation with the loci rs1264427, rs3747676, and rs3789138. Thus, these three SNP loci do not likely participate in the occurrence and development of osteoporosis. Moreover, rs308442 locus do not react with the other loci. Furthermore, LD types by pairs among the most loci in the case and control groups were basically similar.

The degree of LD between loci is not necessarily related to the distance between the loci. Of the two groups of the population, no haplotype block was found. Therefore, the relationship between *FGF-2* polymorphism and osteoporosis cannot be judged through LD analysis results.

## 4. Materials and Methods

### 4.1. Analyses of Bone Density

Firstly, a standard height measuring instrument (HF50, Hengfeng, Chengde, China) was used to measure the net height of subjects. A biological electrical impedance analyzer (MC-180, Tanita, Japan) was used to determine body composition which recorded net body mass, body mass index, and other pertinent data. Achilles Express (GE, Fairfield, IA, USA) was used to perform broadband ultrasound attenuation (BUA) of the right calcaneus of subjects. Prior to ultrasonic BMD detection, 100 samples were chosen randomly, and their BMDs were measured both by applying the dual-energy X-ray absorptiometry (DXA) and quantitative ultrasound methods. After comparison tests, a calibration formula was established, from which a standard module was used for the calibration to ensure the accuracy of the results from the ultrasound method. Bone mass results are represented with BUA, which was based on T-score in decibels per megahertz.

### 4.2. Analyses of FGF-2 Gene Polymorphism

Venous blood (3mL) was extracted from each of the 623 volunteers. After anticoagulation with ethylenediaminetetraacetic acid (EDTA) and conventional proteinase K digestion, the genomic DNA in the white blood cells was extracted using phenol-chloroform method [37], and the DNA sample was stored at −20°C. Prior to use, 1% agarose gel electrophoresis was performed to assess DNA quality and concentration, and DNA samples were diluted to the working concentration of 5–10 ng/μL.

We searched the chromosome location and gene sequence of *FGF-2* gene in the NCBI database and single nucleotide polymorphism (SNP) loci selected in the SNP database. Based on NCBI database, we further searched information about *FGF-2* genetic variation from the International Hap-Map Project database (https://www.genome.gov/10001688/). Haploview 4.2 software was employed to determine the distribution frequency of polymorphic loci. The Tagger function was used to select SNPs with properties of *r*^2^ > 0.8 and minor allele frequency (MAF) > 0.05. As a result, rs308379, rs12644427, rs3789138, and rs308442 loci of the first intron of *FGF-2* gene and rs3747676 locus of the third exon of the 3’-UTR were selected for analyses for the current study. Primer 5 software was used to design primers, which were synthesized by GENESKY (Shanghai Genesky Biotech Co., Ltd., Shanghai, China). The related information on primers is shown in Table 7.

The gene amplification was carried out in PCR reactions of 20 μL, which included 2 μL of 10×buffer, 2.2 μL of MgCl_2_ (25 mmol/L), 0.8 μL of dNTPs (10 mmol/L), 1 μL for each upstream and downstream primers (10 μmol/L), 1 μL of template, 0.2 μL of Taq DNA polymerase, and an appropriate amount of double-distilled water to obtain 20 μL volume. PCR reaction conditions were as follows: 95 °C for 2 min; 11 cycles of 94 °C for 20 s, 65–0.5 °C/cycle for 40 s, and 72 °C for 1.5 min; 24 cycles of 94 °C for 20 s, 59 °C for 30 s, and 72 °C for 1.5 min; 72 °C for 2 min; and 4 °C.PCR products were firstly purified with shrimp alkaline phosphatase (SAP, Promega, Madison, WI, USA) and Exonuclease I (EXO I, Epicentre, Wisconsin, USA) and then were used for the extension reaction with SNaPshot Multiplex kit (Applied Biosystems, Foster, CA, USA). The conditions for extension reaction were as follows: 96 °C for 1 min; 28 cycles of 96 °C for 10 s, 50 °C for 5 s, and 60 °C for 30 s; and 4 °C. The extension product after purification by SAP was sampled on ABI3130xl. SNP genotyping was analyzed with GeneMapper 4.0 (Applied Biosystems).

### 4.3. Linkage Disequilibrium (LD) Analyses on the Five SNP Loci of FGF-2 Gene

SNPs on the same chromosome are not isolated, and SNPs of adjacent allelic genes tend to appear simultaneously. In this study, to analyze the LD of the five SNP loci of the *FGF-2* gene, Haploview 4.2 software was used to detect their D’ and *r*^2^ vales.

### 4.4. Statistical Analyses

Data was represented as x ± SD. The enumeration data was tested using χ^2^-test. Fitting χ^2^ was used to detect whether the gene frequency satisfied the Hardy-Weinberg equilibrium law. PHASE 1.0 software (free software from: http://www.stat.washing ton.edu/stephens/home.html) was used to construct haploids. Odds ratio (OR) values and 95% confidence interval (CI) were used to measure the correlation between polymorphism of *FGF-2* gene and osteoporosis susceptibility. All statistical tests were two-sided probability tests. Sample size was determined using online calculating tools (www.powerandsamplesize.com/Calculators/). *p* Values less than 0.05 indicated statistical significance. SPSS 18.0 software (IBM, Beijing, China) was used for the statistical analyses.

## 5. Conclusions

The current study has shown age-related gradual declines in males and fast and large decreases in females in the calcaneal ultrasound BMD in the senior and middle-aged Zhuang people in Guangxi, China. By analyzing distributions of *FGF-2* gene polymorphism and by exploring correlations between five SNPs of *FGF-2* gene and the BMD in osteoporosis patients and in senior and middle-aged normal bone mass controls, the current study demonstrated that rs308442 locus of *FGF-2* gene is closely correlated to osteoporosis and that the TA may be the risk genotype of osteoporosis. Further studies, such as measurements of serum *FGF-2* levels and bone *FGF-2* mRNA and protein expression levels in both osteoporosis patients and age-matched normal bone mass individuals, are required to prove whether *FGF-2* gene may potentially useful as a genetic marker to predict the risk of osteoporosis in senior and middle-aged Zhuang people.

## Figures and Tables

**Table 1:** Bone mass measurement results as broadband ultrasound attenuation (BUA).

Age Groups	BUA (g/cm^2^)			
	*n*	Males	*n*	Females
45	26	60.03 ± 5.99	14	59.62 ± 5.47
50	66	62.74 ± 4.59	78	61.63 ± 5.44
55	59	61.81 ± 5.52	83	58.02 ± 4.07 **
60	81	60.67 ± 4.66	69	56.62 ± 4.77 **
65	59	59.94 ± 6.01	67	54.69 ± 4.16 **
70	10	58.79 ± 5.17	11	52.58 ± 2.03 **
Total	301	61.09 ± 5.32	322	57.79 ± 5.29 **

Data as x ± SD; ** *p* < 0.01 versus males.

**Table 2:** The age-related changes in numbers and prevalence of individuals with normal bone mass, osteopenia, and osteoporosis.

Age Groups	Normal (*n*, %)		Osteopenia (*n*, %)		Osteoporosis (*n*, %)	
	Male	Female	Male	Female	Male	Female
45	12, 46.15%	6, 42.86%	10, 38.46%	5, 35.71%	4, 15.38%	3, 21.43%
50	43, 65.15%	39, 50%	19, 28.79%	33, 42.31%	4, 6.06%	6, 7.69%
55	30, 50.85%	18, 21.69%	24, 40.68%	44, 53.01%	5, 8.47%	21, 25.3%
60	41, 50.62%	15, 21.74%	28, 34.57%	22, 31.88%	12,14.81%	32, 46.38%
65	24, 40.68%	5, 7.46%	18, 30.51%	19, 28.36%	17, 28.81%	43, 64.18%
≥70	4, 40%	0, 0%	3, 30%	2, 18.18%	3, 30%	9, 81.82%
Total	154, 51.16%	83, 25.78%	102, 33.89%	125, 38.82%	45, 14.95%	114, 35.4%

χ^2^ = 144.653, *p* = 0.000.

**Table 3:** Comparisons of distributions of genotype frequency in the five single nucleotide polymorphism (SNP) loci of *FGF-2* among the Normal, Osteopenia, and Osteoporosis groups.

					Normal vs.Osteoporosis		Normal vs.Osteopenia	
Loci	Genotype	Normal	Osteopenia	Osteoporosis	OR (95% CI)	*p*	OR (95% CI)	*p*
rs12644427	AA	65	59	41	1.000		1.000	
	GA	111	110	81	0.864 (1.403–0.532)	0.555	0.916 (1.422–0.590)	0.696
	GG	61	58	37	1.040 (1.832–0.591)	0.892	0.955 (1.579–0.577)	0.857
Allelic gene								
	A	241	228	163	1.000		1.000	
	G	233	226	155	1.017 (1.351–0.765)	0.909	0.975 (1.262–0.754)	0.849
rs308379	TT	82	84	43	1.000		1.000	
	TA	109	101	82	0.697 (1.112–0.437)	0.129	1.106 (1.661–0.736)	0.629
	AA	46	42	34	0.709 (1.263–0.399)	0.243	1.122 (1.882–0.669)	0.663
Allelic gene								
	T	273	269	168	1.000		1.000	
	A	201	185	150	0.825 (1.097–0.62)	0.186	1.071 (1.39–0.824)	0.609
rs308442	TT	146	124	87	1.000			
	TA	50	84	91	1.652 (1.960–1.424)	0.031	1.653 (1.968–1.441)	0.033
	AA	21	12	8	1.565 (3.690–0.664)	0.303	1.486 (3.142–0.703)	0.297
Allelic gene								
	T	362	339	238	1.000		1.000	
	A	112	115	80	0.92 (1.281–0.662)	0.623	0.912 (1.23–0.676)	0.547
rs3747676	TT	69	70	43	1.000		1.000	
	CT	112	103	85	0.821 (1.319–0.511)	0.415	1.103 (1.690–0.720)	0.652
	CC	56	54	31	1.126 (2.012–0.630)	0.689	1.052 (1.735–0.638)	0.842
Allelic gene								
	T	250	243	171	1.000		1.000	
	C	224	211	147	1.042 (1.386–0.784)	0.776	1.032 (1.336–0.797)	0.811
rs3789138	AA	71	70	43	1.000		1.000	
	GA	116	104	87	0.808 (1.292–0.505)	0.372	1.100 (1.679–0.720)	0.660
	GG	50	53	29	1.044 (1.89–0.577)	0.887	0.930 (1.546–0.560)	0.780
Allelic gene								
	A	258	244	173	1.000		1.000	
	G	216	210	145	0.999 (1.329–0.751)	0.994	0.973 (1.259–0.751)	0.834

**Table 4:** Comparisons of bone mass density in the five SNP loci of *FGF-2* among normal bone mass (Normal), Osteopenia, and Osteoporosis groups.

Loci	Genotype	Normal	Osteopenia	Osteoporosis	Total
rs12644427	AA	64.88 ± 3.84	58.11 ± 1.52	52.85 ± 1.53	59.47 ± 5.51
	GA	65.39 ± 3.52	58.18 ± 1.55	52.67 ± 2.00	59.35 ± 5.69
	GG	64.83 ± 2.97	57.93 ± 1.4	52.51 ± 1.87	59.34 ± 5.36
	*p*	0.505	0.585	0.791	0.791
rs308379	TT	65.02 ± 3.55	58.03 ± 1.48	52.82 ± 2.28	59.17 ± 5.36
	TA	65.29 ± 3.58	58.16 ± 1.53	52.52 ± 1.7	59.24 ± 5.76
	AA	64.82 ± 3.12	58.08 ± 1.49	52.88 ± 1.62	59.70 ± 5.37
	*p*	0.715	0.846	0.545	0.593
rs308442	TT	64.96 ± 3.45	58.11 ± 1.48	52.68 ± 1.98	61.04 ± 5.81
	TA	65.24 ± 3.62	58.03 ± 1.52	52.67 ± 1.72	58.75 ± 5.45
	AA	65.68 ± 3.25	58.48 ± 1.65	52.69 ± 1.62	60.59 ± 5.55
	*p*	0.624	0.630	1.000	0.03
rs3747676	TT	64.83 ± 2.85	57.82 ± 1.44	52.51 ± 1.85	59.67 ± 5.05
	CT	65.57 ± 3.76	58.16 ± 1.52	52.57 ± 1.98	59.34 ± 5.93
	CC	64.53 ± 3.53	58.34 ± 1.52	53.22 ± 1.4	59.22 ± 5.30
	*p*	0.140	0.137	0.191	0.757
rs3789138	AA	64.73 ± 2.87	57.83 ± 1.44	52.51 ± 1.85	59.25 ± 5.27
	GA	65.54 ± 3.7	58.16 ± 1.52	52.6 ± 1.97	59.38 ± 5.91
	GG	64.64 ± 3.65	58.32 ± 1.53	53.15 ± 1.42	59.58 ± 5.09
	*p*	0.167	0.161	0.301	0.873

**Table 5:** Linkage disequilibrium (LD) coefficient D’ in *FGF-2* mutant loci.

Mutant loci	rs308379 Case/Control Group	rs308442 Case/Control Group	rs3747676 Case/Control Group	rs3789138 Case/Control Group
rs12644427	0.144/0.162	0.553/0.524	0.922/0.875	0.966/0.811
rs308379		0.124/0.107	0.182/0.210	0.183/0.174
rs308442			0.238/0.291	0.230/0.214
rs3747676				0.997/0.895

**Table 6:** Linkage disequilibrium (LD) coefficient *r*^2^ in *FGF-2* mutant loci.

Mutant loci	rs308379	rs308442	rs3747676	rs3789138
rs12644427	0.016	0.097	0.725	0.767
rs308379		0.007	0.022	0.021
rs308442			0.021	0.020
rs3747676				0.959

**Table 7:** PCR primers for the five single nucleotide polymorphisms (SNPs) of the *FGF-2* gene.

Polymorphic Loci	Forward Primer (5’–3‘)	Reverse Primer (5’–3‘)	Extension Primer (5’–3‘)
rs12644427	TTCACCATTTATGAAACACTGACTTG	GGGATCATCCAGTACACCTTCCCTAT	TTTTTTTTTTTTTTTTTTTGAAACACTGACTTGTCTGTTTCCA
rs3789138	TCCCTTGCCAATACCTTGTCAT	TTGCAGCCATGTGATTGGTGTC	TTTTTTTTTTTTTTTTTTTTTTTTTTTTTTTTTTTTTTGTGAGTTTTGAGCTAAGTTTTGGAGTA
rs308379	TCCCGTATTTGTTACCTTCTGTCCA	TCCAGCAATTAGGTAGCATGGAGTG	TTTTTTTTTTTTTTTTTTTTTTTTTTTTTTTTTTTTCCAGCCTCATTTAGTCCCCC
rs308442	CCCTTCACGGAATTCCCCAATA	CATCCAGCAAGCATTTATGAAGCAC	TTTTTTTTTTTTTTTTTTTTTTTTTTTTTTTTTTTCAAGCATTTATGAAGCACTCATTG
rs3747676	GGGGACATGCATATTAAGGAAAAGG	TCTCAACTGAGAAATAATCCCCTAACACA	TTTTTTTTTTTTTTTTTTCAAATACATTGATTTGTCATGATACACA
